# Identity Transformation and the Role of Accountability in Recovery from Problematic Pornography Use: A Phenomenological-Hermeneutical Study

**DOI:** 10.3390/jcm15124845

**Published:** 2026-06-22

**Authors:** Luís Lorente-Corral, David Sancho-Cantus, Samuel Asensio, Cristina Cunha-Pérez, Jorge Casaña-Mohedo

**Affiliations:** 1 Doctoral Degree School, Catholic University San Vicente Mártir, 46001 Valencia, Spain; luis.lorente@ucv.es; 2 Department of Nursing, Catholic University San Vicente Mártir, 46001 Valencia, Spain; jorge.casana@ucv.es; 3 Nursing & Mental Health Research Group, Faculty of Medicine and Health Sciences, Catholic University San Vicente Mártir, 46001 Valencia, Spain; 4 Department of Biomedical Sciences, Institute of Biomedical Sciences, Faculty of Health Sciences, Cardenal Herrera, CEU Universities, 46001 Valencia, Spain; samuel@uchceu.es; 5 SONEV Research Group, Faculty of Medicine and Health Sciences, Catholic University San Vicente Mártir, 46001 Valencia, Spain

**Keywords:** problematic pornography use, phenomenology, personal collapse, accountability, support groups, identity transformation, recovery, qualitative study

## Abstract

**Background**: Problematic pornography use (PPU) has emerged as a clinically significant phenomenon with severe repercussions for mental health and interpersonal relationships. Despite advances in prevalence studies, a gap remains in understanding the subjective processes of recovery and personal transformation. **Objective**: To describe the lived experience of individuals in recovery from PPU and compulsive sexual behavior and to analyze the perceived factors and dynamics of group support within their process of change. **Methods**: A qualitative study was conducted following Van Manen’s phenomenological-hermeneutical approach. In-depth semi-structured interviews were held with 27 individuals (26 men, 1 woman) engaged in structured recovery. Data analysis was performed through thematic analysis supported by ATLAS.ti Scientific Software Development GmbH, 2026. **Results**: A structured trajectory was identified across three phases: personal collapse, group engagement, and transformative mechanisms. Accountability emerged as a salient perceived mechanism of change, fostering sincerity (35.7%) and relapse prevention (19.1%). The “mirror effect” and “rational hope” within the support group facilitate a profound identity shift from a “spoiled identity” to a state of personal authenticity. **Conclusions**: For individuals engaged in structured support groups, recovery from PPU transcends mere abstinence, requiring a profound identity transformation facilitated by collective connection and honesty. These findings suggest the potential utility of integrating group-based accountability into therapeutic interventions for behavioral addictions tailored to the patient’s experiential profile.

## 1. Introduction

In the context of today’s hyperconnected society, pornography has transitioned from a marginal consumer product to a globally widespread practice. The “Triple A” model described by Cooper [1] (Accessibility, Affordability, and Anonymity) has radically transformed contact patterns with sexually explicit material, removing entry barriers for the most vulnerable populations and facilitating early exposure, which, according to available clinical data, typically begins during critical adolescence, between the ages of 11 and 16 [2]. This early onset occurs within an environment of profound social normalization—a “backdoor” effect—where pornography consumption is frequently perceived as a harmless recreational activity, which delays both risk awareness and the user’s recognition of their loss of control [3,4].

Available prevalence data confirm the magnitude of this phenomenon across different scales. Globally, a large-scale study conducted in 42 countries—with over 82,000 participants from Europe, North America, Asia, and other regions—estimates that between 3.2% and 16.6% of the adult population may present problematic pornography use (PPU), with a higher prevalence among men than women, noting that this proportion is comparable to other clinically recognized mental health disorders [5]. In Spain, the 2024 EDADES Survey, promoted by the National Plan on Drugs [6], reports that 63.8% of the general population aged 15 to 64 admits to having consumed pornography at least once in their lifetime, with 29.0% declaring use within the last year; among the student population aged 14 to 18 (2023 ESTUDES Survey), 66.8% admit to having used it at least once and 4.1% present indicators of problematic use, a figure that rises to 7.2% among males [7].

From a clinical perspective, problematic pornography consumption manifests as a compulsive pattern with significant consequences for mental health, interpersonal relationships, sexual life, and overall well-being [8,9], with dopaminergic reward mechanisms similar to those observed in substance addictions [10]. Its private and digital nature allows for maintaining an apparent social functionality for years, which delays problem recognition and complicates therapeutic intervention.

Available interventions include cognitive-behavioral approaches and Acceptance and Commitment Therapy (ACT), with moderate empirical support [11], as well as twelve-step mutual aid programs—such as Sex Addicts Anonymous or Pornography Addicts Anonymous—which offer peer support and accountability [12]. Although sustained participation in these groups is associated with lower relapse rates, dropout rates remain high—approximately 40% within the first year [13]—suggesting that group-based approaches may not be optimal for all individuals and underscoring the need to understand the subjective factors that sustain commitment to recovery.

Despite growing scientific interest in this field, research addressing the subjective processes of recovery—and the identity transformations that constitute them—remains scarce [13,14]. This gap is particularly significant given that recovery cannot be reduced to the mere cessation of problematic behavior: sustained change requires deeper transformations in identity, interpersonal relationships, and the individual’s sense of purpose.

This article addresses this phenomenon from three distinct analytical perspectives: (1) the phenomenological trajectory associated with loss of control from initial contact to personal collapse; (2) the processes of identity transformation emerging during the recovery process; and (3) the mechanisms of recovery articulated around accountability and the support group. The integration of these three perspectives allows for a multi-level understanding of the phenomenon, from its origins to its transformative resolution.

To properly frame these objectives, it is essential to establish the conceptual framework of recovery. Contemporary perspectives have moved beyond models focused exclusively on abstinence to include dimensions such as well-being, identity, and social functioning [15,16]. The transition toward a “person in recovery” identity constitutes one of the most relevant changes in the process, as it involves not only modifying behaviors but also transforming the meaning structures through which the individual interprets their experience [14]. From the phenomenological perspective adopted in this study, PPU manifests as an alteration in the structure of the subject’s life-world (Lebenswelt), justifying an approach to recovery from within, by understanding the subjective transformations that sustain it [13,17].

The objective of the present study is to describe the lived experience of individuals with PPU during their recovery process within a support group. Furthermore, it aims to analyze the factors involved in this process of change, as well as to explore the role and dynamics of group-based support as perceived by the participants, in order to gain a deeper understanding of its contribution to the therapeutic process.

## 2. Materials and Methods

### 2.1. Design and Methodological Approach

A qualitative phenomenological-hermeneutical approach [17] was adopted as the guiding framework for this study. Data processing was conducted through thematic analysis [18] using a five-cycle analytical process. This included initial corpus coding, code grouping and category construction, cross-sectional comparative analysis, identification of core themes (metacategories), and the definition of the final analytical model. The choice of a phenomenological-hermeneutical approach responds to the nature of the phenomenon under study; it is grounded in its suitability for addressing complex subjective experiences, distinguishing it from other equally rigorous qualitative methodologies such as grounded theory or ethnographic approaches.

### 2.2. Participants and Data

The study included 27 adult participants (26 men, 1 woman) engaged in a process of self-identified recovery from PPU. The marked gender imbalance in the sample—characterized by a low female representation (3.7%; *n* = 1)—directly reflects the actual gender composition of the active groups at the time of recruitment rather than differential refusal rates. Of the 34 individuals invited to participate, only one was a woman (P25), who accepted; no other women were present in the groups at the time of recruitment. The interview guide did not include a specific question about potential difficulties related to sharing experiences in mixed-sex settings, which constitutes a limitation of the study. Of the groups from which the participants were drawn, one functioned permanently in a mixed-gender format, while the other three, although organized along gender lines, periodically held certain joint activities in a mixed-gender format. Participants joined these groups either through referrals from therapists or by contacting the organization via telephone, driven by their own desire to cease the behavior. Participants were recruited from four separate groups following twelve-step programs for compulsive sexual behavior (such as Sex Addicts Anonymous or Pornography Addicts Anonymous). All participants were informed about the upcoming study and offered, individually (via text message or in-person conversation), the opportunity to participate. An invitation was extended to 34 individuals, of whom 27 ultimately accepted, representing a participation rate of 79.41%. The 7 individuals who declined did not provide reasons for non-participation. These groups have no affiliation with any specific religious denomination, nor do they require membership in any church or faith community. However, like all twelve-step programs, they share a structural theistic base that references a “higher power”, which each participant defines according to their own convictions. This distinction is relevant for interpreting the references to spirituality and transcendence that emerge in the participants’ discourse; they do not reflect institutional religious adherence but rather an existential dimension of meaning that is part of the program’s therapeutic framework. It should be noted that recruiting exclusively from active twelve-step support groups introduces an important selection bias: participants were already committed to a structured recovery process, which likely favors more positive accounts of group-based mechanisms than would be found in a broader population of individuals with PPU. This limitation is discussed further in Section 4.9. All participants were selected through purposive sampling (a non-probability strategy in which participants are deliberately chosen based on specific characteristics relevant to the research question) [19]. Information was gathered until reaching the point where, after hearing a diversity of ideas, no new elements emerged in subsequent interviews— a process known as thematic sufficiency (information power) [20] (Table 1).

Data were collected through face-to-face semi-structured in-depth interviews [21,22]. These were designed to facilitate a chronological narrative structured around three axes: (a) onset and normalization; (b) escalation and consequences; and (c) the moment of crisis or rupture, recovery resources, and perceived personal changes (Appendix A). Interviews were audio-recorded and transcribed verbatim to preserve the fidelity of the discourse and the emotional nuances critical for phenomenological analysis.

The final sample consisted of 27 participants, with a mean chronological age of 34 years (SD = 8.64). The sex distribution showed a marked male predominance (96.3%; *n* = 26), with only one female participant (3.7%; *n* = 1). Regarding employment status, most participants were employed (85.2%; *n* = 23), followed by students (11.1%; *n* = 3) and individuals neither in education nor employment (3.7%; *n* = 1). In terms of marital status, the sample was balanced between married (51.9%; *n* =14) and single individuals (48.1%; *n* = 13). Concerning the frequency of consumption prior to intervention, a daily pattern predominated (51.9%; *n* =14), followed by consumption several times a day (40.7%; *n* = 11) and weekly consumption (7.4%; *n* = 2), reflecting a consolidated and intensive usage behavior in most participants. These data highlight a profile of predominantly male, young-adult, professionally active users with a recurrent consumption pattern—daily or higher in 74.07% of cases—before starting treatment.

Table 2 summarizes the sociodemographic and consumption characteristics of the sample. The participants represent a socially integrated profile (predominantly adult men, professionally active, and in stable relationships) that contrasts with the early age of onset, situated in adolescence. This pattern, together with the high frequency and intensity of consumption recorded in the sample, is of particular relevance from a public health perspective, as it suggests a prolonged and consolidated consumption trajectory according to participants’ retrospective accounts.

Analysis of participants’ prior support trajectories shows that joining a recovery group is seldom their first attempt at help. In many cases, participants had previously sought spiritual guidance, psychological intervention, or informal support. However, these earlier attempts were often perceived as insufficient to resolve the problem, ultimately leading them to seek a more structured recovery environment.

### 2.3. Data Analysis

Data analysis was supported by ATLAS.ti Scientific Software Development GmbH (Berlin, Germany), 2026, following a thematic analysis process. The procedure followed five cyclical phases based on the model by Kalpokaite and Radivojevic [23] (Table 3). (1) Initial coding: data immersion and generation of 200 inductive codes. (2) Grouping and categorization: refining the system to 151 active codes and constructing 9 main analytical categories. (3) Cross-sectional analysis: comparison of patterns and variations across all narratives. (4) Definition of core themes: integration of categories into 4 higher-level metacategories. (5) Analytical model: Construction of a final interpretive model integrating the trajectory from loss of control to personal transformation.

To ensure methodological rigor and research quality, the study aligned with the criteria of Guba and Lincoln [24] and the Consolidated Criteria for Reporting Qualitative Research (COREQ) guidelines (Supplementary Material S2). Credibility and internal consistency were ensured through a reflective three-level cyclical coding process, guaranteeing that findings were firmly grounded in the data.

### 2.4. Ethical Considerations

The present study was conducted in strict compliance with the ethical principles for medical research involving human subjects as established in the Declaration of Helsinki. The research protocol, identified under procedure number UCV/2022-2023/176, received a favorable opinion from the Research Ethics Committee of the Catholic University of Valencia on 13 January 2023. In accordance with the approved protocol, participants’ autonomy was guaranteed by obtaining written informed consent following a detailed explanation of the study’s objectives and characteristics. Data privacy and confidentiality were rigorously protected through the anonymization of testimonies, removing any identifying information during all phases of analysis and utilizing alphanumeric codes for the processing of verbatim accounts. Furthermore, subjects were informed that their participation was strictly voluntary, ensuring the absence of any detrimental effects derived from the study and the right to withdraw at any time without negative consequences.

## 3. Results

**Figure 1:**
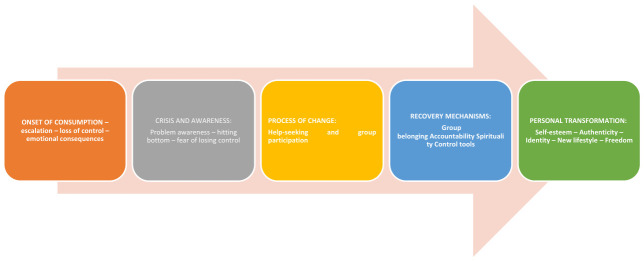
Conceptual model of the recovery process from PPU developed from the qualitative analysis of the interviews.

The qualitative analysis identified and described a trajectory of transformation structured into three major phases: (I) onset of consumption and trajectory toward collapse; (II) the process of change and the emergence of the group; and (III) mechanisms of recovery and transformation. Within these phases, five interconnected core themes are articulated (Figure 1), providing an understanding of the experiences of problematic pornography use, moments of crisis and awareness, recovery itineraries, and the mechanisms that foster personal transformation. The procedure was based on a qualitative analysis model inspired by the cycles proposed by Kalpokaite and Radivojevic and the analytical levels described by Medina Moya, Leyva Moral, and Carrillo Pineda [23,25], adapted to the design of the present study. This model integrates the five core themes identified in the analysis, the nine analytical categories, and the 151 active codes derived from the interview corpus, which emerged from an initial system of 200 codes generated during the open coding phase.

### 3.1. Phase I: Onset of Consumption and Trajectory Toward Collapse

#### 3.1.1. Early Exposure: Normalization and Passive Initiation

The participants’ accounts allow for the reconstruction of a common initial trajectory: the first contact with pornography occurs predominantly during early adolescence, between the ages of 11 and 16, frequently in a non-premeditated manner. Many subjects described that they did not actively seek out pornographic material; rather, they “stumbled upon it” or it was introduced by their peer group within the context of digital socialization (P1, P5, P7, P8, P15, P16).

“…I fell into pornography when I was very young…” (P7) “It was what all my friends did; we didn’t think it was anything bad, it was part of being a man at that time”.

(P8)

#### 3.1.2. The Spiral of Consequences: From Pleasure to Compulsion, Loss of Control, and Problem Recognition

Over time, however, participants described a progressive intensification of consumption, accompanied by the emergence of compulsive usage patterns, loss of control, and difficulties in stopping the behavior. As consumption stabilized, participants described a transition from pleasure to necessity (P9, P16, P12).

“…every day I consumed more pornography, I had access to more things…” (P9) “…I was completely hooked on diving into the internet, it was something I couldn’t stop…”.

(P12)

The narratives show a pattern of escalation—seeking more extreme content to achieve the same effect—and a series of cross-sectional consequences manifested in the following categories:

Identity Deterioration: The emergence of “toxic guilt”. The subject begins to live a double life, which generates a fragmentation of the self.

“I hated what I was doing, but I couldn’t stop. I felt like a hypocrite in front of my family and friends”.

(P14)

Isolation and Relationships: A shift from real desire toward virtual desire is observed. Participants reported a lack of interest in their partners and a preference for the solitude of the screen (P1, P14, P21).

“…the reality is that none of those methodologies worked because, in the end, I found the backdoor [to keep consuming]” (P1) “Pornography became my «refuge» and my «prison» at the same time. I preferred being alone with the computer than going out with real people”.

(P21)

From a phenomenological perspective, this process was characterized by two complementary dimensions that participants described recurringly:

(a) Loss of control: Most accounts agreed that volition was hindered by recurrent consumption, evidencing aspects such as the alteration of self-regulation mechanisms, the compulsive nature of the behavior, and interference in decision-making (P1, P12, P14, P16, P23).

“…I realized that it was no longer me who decided; it was a compulsive need that moved me away from my reality and my values” (P12). “… you wanted to stop masturbation or pornography and so on, and you couldn’t”.

(P16)

(b) Crisis as a Catalyst for Recognition: In several accounts, the recognition of problematic use appeared linked to moments of personal crisis that acted as triggers for change, such as relationship conflicts, emotional exhaustion, or the realization of the inability to quit the behavior by one’s own means. In other cases, awareness occurred through contact with other people who had lived through similar experiences (P5, P6, P11).

“I tried to quit a thousand times on my own, but I always went back. The pain of seeing how it affected my partner was what made me admit I was sick” (P5)....I saw that it affected my relationship with others, that I was very closed off, very cold, and well, I had self-contempt; I felt guilty about some things”.

(P11)

#### 3.1.3. The Myth of Individual Control and the Cycle of Failure and Escape

A key finding in this phase is the struggle to regain autonomy through individual effort. However, the narratives demonstrate that these attempts often reinforce the problematic pattern. The categories identified after reviewing and analyzing the participants’ discourse are shown below:

Broken Promises: The subjects describe cycles of a “final time” that inevitably end in more severe relapses. This constant failure undermines self-efficacy (P6, P16, P21, P23).

“…I started trying to quit pornography…but I didn’t take it that seriously either…”.

(P16)

(b) False Belief of Control: Consumption eventually takes full possession of the individual (P1, P3, P14, P23).

“…I realized at a given moment that many times I didn’t want to do it, and I couldn’t say no to myself; I couldn’t stop doing it, at least not by my own strength…” (P14). “…the idea that destroyed me…was thinking that I already had it under control…”.

(P23)

#### 3.1.4. Personal Collapse as a Turning Point

The recognition of the problem was not the result of rational reflection but rather a systemic collapse. Participants identified turning points where “normalcy” definitively broke down. The following were the emerging categories:

Catalytic Events: Relationship crises, health issues (such as porn-induced erectile dysfunction), or the risk of losing employment were common triggers (P6, P10, P11).

“…pornography has not allowed me to have a commitment to my wife and a clean, healthy relationship with her…” (P6). “…I had a girlfriend, so there was also the issue of how I treated her and sought her out in that more sexual sense”.

(P11)

Hitting Bottom: This moment is described as a “surrender” (P3, P4, P8, P10, P27).

“…when one has hit bottom and is willing to do anything to get out of the problematic behavior” (P4). “…there was a moment when I hit bottom, where I didn’t even know where I stood, where on a human and also spiritual level, I thought my life was not worth living “ .

(P8)

### 3.2. Phase II: The Process of Change and the Emergence of the Group

#### 3.2.1. The Support Group: From Loneliness to Collective Connection

Discourse analysis positions the group as the backbone of the recovery process, serving as an essential space for support, belonging, and companionship. The centrality of this construct is further validated by the quantitative analysis of the corpus (26,527 words), where the term “group” emerged as the most frequent, appearing *n* = 683 times (2.58% of the total). This lexical preeminence, combined with the recurrence of concepts such as “recovery” (*n* = 385; 1.45%), “help” (*n* = 226; 0.85%), and “process” (*n* = 216; 0.81%), consolidates the vision of a progressive and sustained therapeutic change, in full alignment with the participants’ narrative trajectories.

The relational dimension of recovery is manifested in the high frequency of terms linked to otherness, such as “person”, “people”, and “others” (grouped frequency of *n* = 484), providing evidence that identity reconstruction is necessarily developed through interaction with others. This community component is intertwined with the sphere of intimate experience, represented by the significant presence of the terms “problematic use” (*n* = 210; 0.79%) and “life” (*n* = 175; 0.66%).

Finally, the findings reveal a positive reframing of the lived experience. While terms such as “problem” (*n* = 139) and “important” (*n* = 137) reflect an awareness of the severity of the disorder, the recurrent use of “better” (*n* = 154) and “hope” (*n* = 146) points toward an optimistic projection of the future. This transformative framework is completed by cross-sectional references to “spirituality” and transcendence—elements that act as a fundamental support in a discourse where recovery integrates group support, individual commitment, and existential meaning.

The results demonstrated a clear predominance of group support as a central element in the recovery process as narrated by these participants. The group was associated with the cessation of consumption, the maintenance of change, and the generation of bonds based on identification and equality among its members. Furthermore, it provided continuous and practical accompaniment that fostered relapse prevention. Individual accompaniment was described as relevant for self-knowledge, understanding the problem, and addressing deeper aspects that were not always reached within the group, and could serve as a complementary resource depending on individual needs. It should be noted that this comparison reflects participants’ subjective perceptions within a group-based program, and does not constitute evidence of the relative efficacy of group versus individual therapy.

#### 3.2.2. The Support Group: Mirror Effect, Rational Hope, and Collective Strength

Participants expressed a clear perception of the distinct dynamics of group support in comparison to prior. The research identifies three analytical categories within the group dynamics.

The “Mirror Effect”: By seeing themselves reflected in the stories and struggles of others, participants manage to reduce resistance to treatment and rebuild their self-esteem. The experience of unconditional, non-judgmental acceptance within the group is described as transformative (P22, P19).

“In the group, I didn’t feel judged; I felt understood. That collective strength gave me the momentum that my individual willpower lacked” (P22).” “Recovery is not a solitary recovery; it is a recovery with people”.

(P19)

“Rational Hope”: Interaction with peers who have achieved significant periods of sobriety offers empirical evidence that recovery is possible, transforming the perception of being “condemned” into motivation for change. The group thus operates as a reservoir of hope (P1, P4, P8, P13).

“I saw that when I joined, there were people who had been without consuming longer than me…and I said, well, I want that too” (P1). “…in the end, when you are in addiction, you are blind, and so it is not easy to have that hope and say, ‘I’m going to get out of here…’”.

(P4)

“Collective Strength”: The group acted as a necessary substitute for individual will, which had been exhausted after multiple cycles of failed attempts. The relational dimension of change—the group as a context that makes individual transformations possible—emerged as one of the core findings of the overall research (P1, P3, P6, P8).

“…recovery truly begins the day I walk into the group” (P1) “…my recovery process began upon entering the group …”.

(P3)

#### 3.2.3. Breaking the Isolation

Participants described PPU as a phenomenon marked by secrecy and isolation. Group participation allowed for the breaking of this dynamic by providing a space for understanding and peer support among individuals undergoing similar experiences. The normalization of the experience—discovering that “one is not alone”—reduced shame and facilitated emotional openness (P1, P5, P8, P9, P14, P17, P19).

“…I have had moments where…it was very hard for me to tell the truth, but when I told it, I felt a great sense of peace” (P8). “…it didn’t start to heal until I brought it to light”.

(P14)

#### 3.2.4. Social Learning and Modeling

Participants reported that observing peers who had been in recovery for longer periods and had successfully maintained abstinence served as a powerful mechanism for learning and motivation. Seeing others overcome what seemed impossible to them expanded their horizon of possibilities and reinforced their belief in their own capacity for change (P1, P5, P12, P17).

“…I have also made a commitment to the others…” (P5). “…seeing that other people had been able to…gave me back my hope”.

(P12)

#### 3.2.5. Positive Moral Pressure

The presence of the group generated, according to the participants, a form of social responsibility experienced as motivating rather than coercive. The desire not to betray the trust of their peers, to uphold the commitment made, and to be able to “reach the next meeting and say I was able to overcome that temptation” acts as an external behavioral regulator that is progressively internalized as personal motivation (P3, P6, P10, P12, P21, P23).

“…I am not going to do this because if I do, I will have to tell it…or it will have implications…” (P10). “…I cannot fail the team”.

(P23)

### 3.3. Phase III: Recovery Mechanisms and Personal Transformation

#### 3.3.1. Recovery Mechanisms

The discourse analysis allowed for the identification of a series of mechanisms that make these transformations possible.

##### Verbalization and Externalization of the Impulse

One of the mechanisms most consistently highlighted by the participants is the ability to verbalize within the group the impulses, thoughts, and problematic behaviors that had not been shared with anyone until that point. The externalization of the impulse—the act of “getting it out” or “saying it out loud”—allows the individual to distance themselves from the problem, reduce its intensity, and avoid dynamics of self-deception (P1, P2, P4, P13, P16, P19, P20, P21).

“The first thing was getting it out; I would get it out, whether through a call with one of the people from the group or via the group chat we had. It helped me to push it out, to say it out loud” (P1). “The face-to-face meetings have been key because every time you come to an in-person meeting, you leave with a lot of motivation” (P2). “Having to report three times a day is fundamental—it keeps you on edge and helps you know that there are people fighting like you at that very moment” (P4). “The challenge that helped me most was the one my older brother set for me” (P13). “Phone calls have helped me—I think they have been vital for me”.

(P16)

Participants described a range of concrete practical tools through which verbalization was operationalized in daily recovery. These included structured daily check-ins with a sponsor or accountability partner, the use of group messaging platforms to externalize urges in real time before acting on them, attendance at emergency or unscheduled meetings during high-risk moments, and the use of postponement techniques—committing to wait a defined period and contact a group member before engaging in the problematic behavior. Several participants described how the simple act of sending a message or making a call was sufficient to defuse the impulse: the externalization itself, rather than the response received, appeared to be the active ingredient (P1, P4, P16, P20).

“Knowing I would have to report it at the next meeting was enough to make me stop and think”.

(P4)

This mechanism aligns with the foundations of narrative therapy and the cognitive-behavioral approach, both of which have highlighted the role of verbalization in emotional regulation and the deactivation of automatic schemas.

##### Accountability and Behavioral Regulation

Accountability constituted, in the participants’ discourse, one of the pillars of the change process. The knowledge that one’s actions must subsequently be shared with the group acts as a self-regulation mechanism that helps curb high-risk behaviors. This mechanism operates at two levels: as an “anticipatory brake” against the impulse, and as a reinforcement of commitment to one’s own values (P1, P2, P3, P4, P5, P7, P12, P16, P18, P22, P25).

“…having to be accountable to others in the group means I have to be true to what I experience and what I do” (P2). “The fact of being in a group where every day you have to report how you’ve lived through things…if I didn’t have to tell anyone what I’m doing, I would keep doing whatever I felt like” (P4)....Accountability was a liberation for me; for me, it was finally being able to be sincere”.

(P7)

Also promoted honesty and transparency, countering the dynamics of concealment inherent to the problematic usage behavior. Several participants described this process as a transition from “living in a lie” to “living in the truth” (P2, P14).

“…It has meant starting to live in the truth, that is, starting to live in accordance with how I want to live” (P2). “My problem was the secrecy…Accountability is what saved me, bringing it to light”.

(P14)

Table 4 summarizes the perceived consequences of Accountability, expressed as the frequency of occurrence within the participants’ discourse. The results reveal a consistent pattern: the most significant consequences are those linked to cognitive-emotional processes—sincerity and honesty (35.7%), relapse prevention (19.1%), and personal responsibility (11.9%)—suggesting that Accountability acts primarily as a self-regulation mechanism. To a lesser extent, dimensions related to verbalization and self-knowledge emerge, pointing toward a metacognitive effect of the therapeutic process on problematic behavior.

#### 3.3.2. Personal Transformation

##### Reconstruction of Self-Esteem

One of the most consistently described changes by participants was the progressive transformation of their self-perception. At the beginning of the recovery process, many reported profound feelings of guilt, shame, and self-loathing associated with their consumption. Throughout the process, a gradual decrease in these emotions is observed, alongside the development of a more positive self-valuation. This shift is linked both to the experience of being accepted without judgment within the group and to the practice of personal honesty (P1, P3, P7, P8, P15, P16, P18, P20, P25).

“When I first joined…I was unable to look other men in the eye…[then] starting to look them in the eyes…feeling like just another one of them” (P7) “…recovering my self-confidence…”.

(P25)

These testimonials suggest a process of emotional reconstruction in which self-esteem is not merely a correlate of cessation of consumption, but one of its primary drivers. The experience of acceptance within the group, the reduction of guilt, and the development of a more compassionate internal relationship appear as necessary conditions for personal transformation. Furthermore, several participants described the development of self-love and self-acceptance as core dimensions of the process (P2, P9, P21).

“…Starting to accept myself and forgiving myself for many things from my past” (P9). “It is about finding self-love”.

(P21)

The overcoming of self-deception also appears to emerge as an essential component of this dimension. The group and the practice of accountability allow participants to become aware of the cognitive distortions that sustained the problematic behavior and to move toward greater personal honesty.

##### Development of Personal Authenticity

The recovery process involved a progressive approach toward one’s own personal truth. Participants described an evolution from lifestyles marked by inconsistency, concealment, and a “double life” toward greater sincerity with themselves and others. This process materialized in the capacity to recognize themselves and act consistently with their own values and experiences (P2, P3, P7, P16, P25).

“…to be able to speak in truth now…” (P16). “…to express and say with freedom the things that I thought in any aspect”.

(P25)

Abandoning the “double life”—one of the most recurrent characteristics in the testimonials—becomes a liberating process. Transparency, coherence between values and behavior, and the genuine expression of the self emerge as indicators of a progressively consolidated personal authenticity.

##### Recovery of Personal Identity

The testimonials verbalize that the recovery process implies a profound reconstruction of personal identity. Participants describe a transition from a self-perception marked by dependency, disorganization, and incapacity toward a more integrated, autonomous, and coherent identity. This process manifests in the rediscovery of themselves, in the development of new ways of understanding their lives, and in the consolidation of a more stable self (P2, P16, P17, P18).

“Recovering my life…not just surviving but recovering my life” (P17). “…for me, it is a new stage beginning…a new rebirth because it is the rest of my life…”.

(P18)

The recovery of identity also implies a change in how they understand themselves in relation to others. Several participants described a relational transformation that includes the capacity for self-giving, commitment, and establishing bonds based on trust and authenticity (P3, P19).

“…I can live, give myself completely to my future wife, give myself completely to my parents, to my friends, that I can give myself completely to God” (P3). “…being able to give of oneself…to form a union…to generate love…”.

(P19)

The process is also characterized by a shift in the sense of self: participants move from seeing themselves as victims of the problematic use pattern to recognizing themselves as active protagonists of their own change process.

##### Adoption of New Lifestyles

Participants described how recovery translated into a profound reorganization of daily life. This transformation went beyond the cessation of consumption and involved the incorporation of healthier habits, greater emotional stability, the recovery of abandoned routines, and a more balanced way of living oriented toward the present (P2, P7, P8, P10, P18).

“It is being able to start leading a normal life that I couldn’t lead before” (P2).”For me, it is a new stage beginning…a new rebirth because it is the rest of my life”.

(P18)

##### Recovery of Personal Freedom

Freedom emerges in the testimonials as the ultimate horizon of the transformation process. Participants describe the problematic usage pattern as a form of internal slavery that compromised their ability to decide and act with autonomy. The recovery of freedom appears as the experience of becoming masters of themselves again (P2, P3, P4, P5, P7, P17, P25).

“Well, I understand recovery as…being able to be free…” (P5). “…it is being able to take back the reins of my life and to be free, with total freedom to choose what I want to do”.

(P7)

The recovery of freedom was described by participants not merely as impulse control, but as the reclaiming of the capacity to choose, to commit, and to live with coherence. This process was supported by the development of concrete self-regulation tools, group commitment, and the progressive construction of a more solid identity.

## 4. Discussion

The results of the present study provide an understanding of PPU that goes beyond clinical symptomatology, underscoring the importance of phenomenological, relational, and existential determinants in both the genesis and resolution of the disorder. The following sections discuss the central findings in relation to the scientific literature and their clinical implications.

### 4.1. The “Backdoor” Phenomenon and Social Normalization as Systemic Risk Factors

The results underscore that early initiation—between ages 11 and 16—was perceived by the participants as a significant factor in their prolonged consumption trajectory. While the retrospective and qualitative nature of this study does not allow for a causal link to be established regarding chronicity, the narratives suggest that early exposure complicates initial risk awareness. This finding is consistent with neurocognitive development theories that warn of the reward system’s vulnerability during adolescence [10]. An original contribution of this research is the conceptualization of the “backdoor” as an entry mechanism: the perception of consumption as an innocuous and socially accepted activity that fails to trigger danger alarms until problematic use is consolidated. This normalization contrasts with problematic use of illegal substances and explains why the subject only recognizes the problem once the relational or sexual damage is already devastating. The implications for prevention are evident: the health system and social support networks cannot afford to wait for total collapse to occur before intervening. Understanding the prior trajectory allows for the design of earlier, more effective interventions.

A finding of particular relevance in this line of inquiry is what this research terms the “backdoor”, referring to the initial perception of consumption as an innocuous and socially accepted activity. Unlike illegal substances, where a clear moral and legal barrier exists, pornography is presented as a “free-access cultural artifact”. This normalization generates a state of anomie in the subject, who lacks the reference points to identify when recreational use transforms into a pathological state. It is important to clarify how this “backdoor” mechanism differs from related concepts: whereas social normalization refers to a broad cultural process by which pornography consumption becomes collectively accepted, the “backdoor” as conceptualized here denotes a specifically individualized cognitive mechanism—the subjective framing by which the user perceives pornography as a culturally legitimate, morally neutral artifact, thereby preventing the formation of internal alarm thresholds. This is distinct from low perceived risk (an external, context-dependent attribution) and from delayed problem recognition (a temporal observation about when help is sought); rather, it captures the active self-exemption from problematic-use categories that allows continued consumption without self-identification as someone with a problem.

Stigma does not emerge during consumption but rather with problematic behavior, trapping the individual in a paradox: they consume what society promotes, yet suffer in silence the consequences that society ignores. These results resonate with Goffman’s [26] framework on the management of spoiled identities and with clinical evidence regarding diagnostic delay in behavioral patterns [8,27].

### 4.2. Toxic Guilt and the Double Life: Spoiled Identity as a Problematic Drive

A central axis of this discussion is the fragmentation of the “self” experienced by individuals with PPU. The transition from curiosity to compulsion is not merely a behavioral change but a profound identity crisis. The “toxic guilt” identified in the corpus differs fundamentally from functional guilt: while the latter drives change, toxic guilt generates emotional paralysis that feeds back into consumption as an escape mechanism. From Goffman’s perspective [26], the management of a “spoiled identity” in the shadows exhausts the subject’s cognitive resources, leading to social isolation that, paradoxically, pushes them back to the screen as their only “safe refuge”. Our data confirm that isolation is not merely a consequence of problematic use but rather the engine that keeps it active. This necessitates that interventions explicitly address the identity dimension and not just the behavioral one.

In this sense, the data from the present study confirm that PPU generates stigma and a “toxic guilt” that only appears to dissolve through peer recognition. This finding is consistent with the work of Tangney et al. on moral emotions and behavior, which distinguishes between adaptive guilt—which drives change—and toxic guilt—which generates paralysis and feeds back into problematic use patterns [28]. The support group, therefore, emerges not only as a clinical resource but as the unique social space capable of dissolving this stigma, in line with Grubbs et al. regarding the role of moral incongruence in problematic pornography use [9].

### 4.3. Accountability as an Antidote to the Secrecy Circuit

A central finding of this research is how participants experienced accountability as a particularly salient self-regulatory resource for interrupting problematic usage behavior. While classical literature often emphasizes individual self-control strategies [29], our results suggest that the “externalization of the impulse” is the differentiating factor that deactivates the strength of desire. By verbalizing the impulse to a peer before acting, the subject manages to create a “safety gap” between the stimulus and the response, causing the desire to lose its emotional charge almost instantaneously. This mechanism connects with the foundations of narrative therapy and the cognitive-behavioral approach, which have highlighted the role of verbalization in emotional regulation and the deactivation of automatic schemas [30]. Accountability acts, ultimately, as an antidote to digital ostracism, allowing the individual to emerge from isolation and strengthen their commitment to sustained behavioral change over time.

### 4.4. The Group as a Therapeutic Mirror and Context of Transformation

The clear preference for group support over individual approaches, as expressed by participants already engaged in twelve-step programs, suggests that human connection was experienced by these individuals as more than emotional support—it functioned as a relational context that made individual transformation possible. This finding should not be interpreted as evidence of the superiority of group therapy over individual approaches such as CBT or ACT, for which stronger controlled evidence exists; rather, it identifies the specific within-group mechanisms that these participants found salient in their recovery. The “mirror effect” acts as a validation device that reduces treatment resistance and reconstructs self-esteem. The group also operates as a reservoir of “rational hope”: interaction with peers who have achieved significant periods of sobriety offers empirical evidence that recovery is possible. This community dynamic provides a “collective strength” that functions as a necessary substitute for individual willpower. The five identified mechanisms of change—impulse verbalization, accountability, breaking isolation, social learning, and positive moral pressure—operate fundamentally within the relational space of the group, which has direct implications for the design of interventions [12,30,31].

These findings suggest that recovery constitutes a life-building project in which participants do not merely leave problematic behavior behind, but actively redesign their purposes, priorities, and ways of relating to their environment. This understanding is consistent with recovery capital models [15] and with studies emphasizing the importance of structured social support in maintaining long-term abstinence [12,31]. The findings of the present study also offer an interpretive reading of the high dropout rates documented in 12-step programs—estimated at approximately 40% during the first year [13]. Disengagement from the group implies the simultaneous loss of all the identified mechanisms of change (verbalization, accountability, the mirror effect, social learning, and positive moral pressure), suggesting that improving group retention should constitute a clinical priority in the design of these programs. At the same time, these high dropout figures are a reminder that group-based approaches are not optimal for all individuals with problematic pornography use: individual therapy, including CBT and ACT, remains an important and empirically supported alternative for those who disengage from or do not benefit from group formats.

### 4.5. Spirituality and the Transformation of the Self

The research highlights that effective recovery should not be understood simply as abstinence, but as a multidimensional process of reconstructing identity and personal freedom. The integration of spirituality and hope emerged, for these participants, not only as a set of beliefs but as a meaningful framework that supported sustained transformation. Whether this dimension operates similarly outside twelve-step or spiritually-oriented contexts remains an open empirical question. This perspective allows the individual to transition from a stigmatized identity of a “struggling user” toward one based on integrity, facilitating self-forgiveness and an objective view of one’s own wounds. These findings are consistent with the work of McIntosh and McKeganey and White, who underscore the importance of constructing a “recovery narrative” as a determining factor in the sustainability of change. Recovery is experienced not as a return to a previous state, but as the construction of a new, more authentic, and freer way of life [13,14].

### 4.6. The Uniqueness of the “Digital Drug”: Comparison with Other Addictions

Unlike substance addictions, PPU is classified under behavioral addictions, sharing neurobiological reward mechanisms analogous to those of pathological gambling [27]. However, it presents unique sociological nuances: perpetual availability and zero cost break traditional impulse control mechanisms; the deeply private nature of the problematic usage pattern allows the subject to maintain social functionality for years while their internal world collapses; and the absence of a moral or legal barrier means the individual only recognizes the problem when relational or sexual damage is already devastating. These factors render intervention strategies designed for other addictions insufficient and underscore the need for specific models for this phenomenon.

### 4.7. Gender Perspective in Recovery: The Case of Participant P25

Although the sample is predominantly male, the inclusion of a single female participant (P25) does not permit comparative statements about gender differences. The account of P25 is retained here as a single-case illustrative observation. P25 described a recovery process in which rebuilding self-esteem and confronting the shame of concealment were central themes. These observations do not support generalizations about women’s experiences of PPU or recovery, and should be interpreted solely as a direction for future research. Future studies should aim to recruit more balanced samples in terms of gender in order to explore whether mechanisms such as accountability and group support operate differently across genders.

### 4.8. Contrast with Existing Literature

The findings of the present study show a highly positive valuation of group support and twelve-step programs as the central axis of recovery. However, this conclusion is not unanimous in the literature.

First, regarding the efficacy of twelve-step programs for compulsive sexual behavior, a recent systematic review identified only eight empirical studies on the use of the twelve-step method in this domain and Compulsive Sexual Behavior Disorder (CSBD), of which only three were considered of high methodological quality; the results were inconclusive, and the authors conclude that the available evidence does not allow for a solid assertion of the superiority of this approach as a standalone treatment, noting that the most favorable data point toward a combination of group support and individual therapy [32]. This limitation contrasts with the clearly positive perception of the participants in the present study, which could be explained by the selection bias inherent to the design: participants already active in the group and motivated for change offer more elaborate transformation narratives than those an experimental trial with a more heterogeneous sample might detect [33].

Second, in contrast to the centrality that the present study grants to group support, other research has highlighted that individual therapy—particularly CBT and acceptance and commitment therapy—presents greater empirical support as a primary intervention. A recent meta-analysis on psychotherapy for problematic pornography use, which integrated randomized controlled trials and non-controlled studies, concluded that both CBT and ACT produce significant and sustained reductions in problematic use, craving symptoms, and sexual compulsivity, with effects maintained at follow-up [11]. These results suggest that the individual component may be, in some contexts, more determinant than the group component—which does not deny the utility of the group, but does relativize its role as the “central axis” and irreplaceable element of recovery, as perceived by the participants of this study.

Third, there is a debate in the literature regarding the possible iatrogenic effects of programs based on the problematic consumption model. From critical positions, it has been argued that the addiction-focused framework may reinforce shame, intensify sexual aversion, and generate greater distress in individuals whose behaviors are—from the perspective of contemporary clinical sexology—variants of normality or expressions of moral incongruence rather than problematic behavior per se [9,34]. Furthermore, the incorporation of the “higher power” concept in twelve-step groups, although defined in non-confessional terms within these groups, can generate resistance or discomfort in participants without spiritual reference frames, which could contribute to the high dropout rates documented in the literature [13].

Finally, in relation to identity transformation as the core of recovery—one of the most consistent findings of the present study—the literature offers a more complex perspective. Although the construction of a “recovery narrative” and the adoption of a new identity have been linked to greater sustainability of change [13,14], other studies indicate that excessive identification with the label of “recovering user” can hinder social reintegration and reinforce group dependency dynamics that, paradoxically, limit the autonomy that recovery aims to restore [15,35]. These tensions do not invalidate the findings of the present study, but they underscore the need to understand group support as a transitional tool that must evolve toward the reinforcement of individual self-efficacy, rather than as an end in itself.

### 4.9. Limitations and Future Research Perspectives

Despite the methodological rigor and having achieved thematic sufficiency between participants 24 and 27, the present study is not without limitations that must be considered when interpreting its findings. Firstly, due to the qualitative and interpretative nature of the design, the results obtained from the sample of 27 subjects do not allow for statistical generalization to the global population with problematic pornography consumption. The analyzed discourses reflect deep subjective experiences but are situated within a specific context where spirituality and faith emerged as fundamental frameworks of meaning for identity transformation. This particularity of the sample could limit the transferability of the conclusions to strictly secular environments or to individuals who do not recognize a transcendent dimension in their recovery process.

Likewise, there is a risk of biases inherent to the use of semi-structured interviews and verbatim accounts. As participants were at different stages of recovery, they may have incurred memory bias or a degree of social desirability when narrating their transition from isolation toward “radical transparency.” On the other hand, since the analysis focused primarily on the role of the support group and accountability as key tools, other concurrent clinical interventions that might have influenced the success of individual treatment were not explored in depth, which restricts the evaluation of the isolated efficacy of each mechanism.

Looking ahead to future research, it is imperative to employ mixed-methods approaches that allow for the quantification of the efficacy of accountability in relapse prevention across more diverse populations. Furthermore, it is necessary to explore in greater depth the impact of early exposure to sexual content—identified between the ages of 11 and 16 in this study—on the chronicity of problematic use and the response to community recovery models. Finally, it is recommended to broaden the analytical spectrum to evaluate how the “collective strength” of the group interacts with traditional cognitive-behavioral therapies in different sociocultural contexts where the normalization of pornography consumption varies significantly.

A further limitation concerns the study’s inability to determine whether social isolation preceded the onset of pornography use or was primarily a consequence of it. The interview guide focused on experiences from first exposure onwards and did not include retrospective screening for pre-existing loneliness or social withdrawal. Future studies should incorporate such screening in order to clarify whether isolation constitutes a vulnerability factor that predisposes individuals to problematic use, or whether it emerges primarily as a consequence of the problematic use process itself. It must also be noted that there is a selection bias inherent to the study design: all participants were recruited from within active support groups, implying that these are individuals who had already taken the step of seeking structured help and who, presumably, presented a higher level of motivation for change than the general population with problematic pornography consumption. This specific profile—committed to recovery and with access to a support network—may have favored the collection of more elaborate and positive transformation narratives, which limits the transferability of the findings to individuals who, while presenting problematic consumption, are not linked to any support group or have not initiated a formal recovery process. Future studies should incorporate more diverse samples in terms of motivational stages and coping modalities, in order to contrast whether the identified mechanisms—accountability, group support, identity transformation—operate analogously in contexts of unstructured recovery or low adherence.

## 5. Conclusions

The findings of this study allow for the formulation of the following conclusions of a theoretical and practical nature.

First, PPU is a systemic and multidimensional process, not an isolated event. The trajectory toward collapse is profoundly mediated by an environment of sociocultural normalization that vanishes risk during the initial stages of consumption, and the “backdoor” constitutes an invisible entry mechanism that imperceptibly transforms curiosity into a compulsive pattern.

Second, personal collapse—far from representing ultimate failure—constitutes the necessary catalyst for seeking help. Intervention strategies should seek to anticipate this tipping point and work to reduce the damage in the familial and mental health dimensions that precede it.

Third, community connection and radical transparency are the critical determinants for sustained change. Faced with the systematic failure of individual self-control strategies, the tool of accountability was experienced by participants as a particularly salient self-regulatory resource for interrupting the secrecy circuit, while group support provides the “mirror effect”, “rational hope”, and “collective strength” necessary to dissolve toxic guilt and stigma.

Fourth, recovery implies an integral reconfiguration of the “self”, articulated across five core dimensions: reconstruction of self-esteem, development of personal authenticity, recovery of identity, adoption of new lifestyles, and the reclaiming of personal freedom.

This understanding of recovery as transformation—and not merely as abstinence—offers a potentially valuable horizon for therapeutic intervention in this field, particularly for individuals engaged in structured group-based recovery programs.

Finally, the findings of this study suggest that public health programs combining structured accountability, peer support, and sensitive attention to existential and meaning-making dimensions of the individual may represent a promising avenue worth exploring in future intervention development. Clinicians are encouraged to assess whether spiritual or existential resources are personally meaningful to individual patients, integrating them when appropriate rather than as universal components. It must be acknowledged that these findings arise from a sample recruited exclusively from groups with a theistic structural basis (twelve-step programs referencing a “higher power”), which limits their generalisability to populations for whom spiritual or transcendent frameworks are not personally relevant. Recovery is not, ultimately, a return to a previous state, but rather the construction of a new, more authentic, and freer way of life.

## Figures and Tables

**Table 1:** Code sufficiency process during interview analysis.

Analyzed Interviews	Generation of New Codes	Analytical Observation
P1–P3	Very High	Initial coding system related to the onset of consumption, guilt, spirituality, group belonging, and accountability.
P4–P6	High	Codes expanded regarding emotional consequences, personal crises, and initial failed attempts to control consumption.
P7–P10	Moderate	Emergence of codes related to group tools, community dynamics, and experiences of acceptance.
P11–P14	Moderate	Consolidation of codes on hope, spirituality in recovery, and reconstruction of self-esteem.
P15–P18	Low	New data reinforce existing codes and provide minor variations regarding program tools and the recovery process.
P19–P23	Very Low	Few new codes emerge; interviews confirm the analytical patterns already identified.
P24–P27	Very Low/Residual	Specific codes appear that nuance processes such as relapse, spirituality, or group experience, but no new analytical categories are generated, indicating stabilization of the analysis system.

**Table 2:** Sociodemographic and consumption characteristics of the sample (*n*= 27).

Variable	Category	*n*	%	M (SD)
Sex	Male	26	96.3	—
	Female	1	3.7	—
Marital Status	Single	13	48.1	—
	Married	14	51.9	—
Employment Status	Student	3	11.1	—
	Employed	23	85.2	—
	Neither studying nor working	1	3.7	—
Consumption Frequency	Weekly	2	7.4	—
	Daily	14	51.9	—
	Several times a day	11	40.7	—
Continuous variables				
Chronological Age		—	—	34.00 (8.64)
Age of Onset		—	—	13.56 (4.90)
Age of Recurrent Use		—	—	18.00 (6.47)
Minutes per Session		—	—	58.70 (41.52)
Prior individual support (before joining the group)				
Type of individual support received	No prior individual support	9	33.3	—
	Psychological support	11	40.7	—
	Psychiatric support	6	22.2	—
	Spiritual accompaniment	11	40.7	—
	Informal support only	3	11.1	—
	Support unrelated to PPU	2	7.4	—
Perceived efficacy of prior individual support *	No efficacy	4	14.8	—
	Partial efficacy	8	29.7	—
	Effective	2	7.4	—
	Not specified/not applicable	13	48.1	—
Duration of prior individual support	<1 year	4	14.8	—
	1–2 years	5	18.5	—
	>2 years	4	14.8	—
	Not specified/not applicable	14	51.9	—
Variable	Category	*n*	%	M (SD)
Sex	Male	26	96.3	—
	Female	1	3.7	—
Marital Status	Single	13	48.1	—
	Married	14	51.9	—
Employment Status	Student	3	11.1	—
	Employed	23	85.2	—
	Neither studying nor working	1	3.7	—
Consumption Frequency	Weekly	2	7.4	—
	Daily	14	51.9	—
	Several times a day	11	40.7	—
Continuous variables				
Chronological Age		—	—	34.00 (8.64)
Age of Onset		—	—	13.56 (4.90)
Age of Recurrent Use		—	—	18.00 (6.47)
Minutes per Session		—	—	58.70 (41.52)
Prior individual support (before joining the group)				
Type of individual support received	No prior individual support	9	33.3	—
	Psychological support	11	40.7	—
	Psychiatric support	6	22.2	—
	Spiritual accompaniment	11	40.7	—
	Informal support only	3	11.1	—
	Support unrelated to PPU	2	7.4	—
Perceived efficacy of prior individual support *	No efficacy	4	14.8	—
	Partial efficacy	8	29.7	—
	Effective	2	7.4	—
	Not specified/not applicable	13	48.1	—
Duration of prior individual support	<1 year	4	14.8	—
	1–2 years	5	18.5	—
	>2 years	4	14.8	—
	Not specified/not applicable	14	51.9	—

Note. M = mean; SD = standard deviation; — = not applicable. Percentages may exceed 100% in the support-type rows because participants could receive more than one type of support simultaneously. * Based on participants who received prior individual support (*n* = 18). For individual participant details, see Supplementary Table S1.

**Table 3:** Evolution of the study’s coding system.

Phases of Analysis	Number of Codes	Process Description
Initial open coding	200	Inductive identification of units of meaning based on a detailed reading of the interviews. Preliminary codes were generated to capture experiences related to consumption, emotional consequences, problem recognition, coping strategies, and recovery processes.
Code review and refinement	188	Constant comparison process between codes to identify redundancies, conceptual similarities, and semantic relationships. In this phase, similar codes were merged, and some excessively specific or repetitive codes were removed.
Final coding system	151	Consolidation of the final analytical system. These active codes group the units of meaning used in the interpretative analysis of the study and are subsequently organized into analytical categories and thematic cores.

**Table 4:** Perceived consequences of accountability in the recovery process.

Identified Consequences	Frequency	Percentage
Sincerity—Honesty	15	35.7%
Relapse Prevention	8	19.1%
Personal Responsibility—Commitment	5	11.9%
Greater Awareness of the Problem	4	9.5%
Transparency	4	9.5%
Externalization of problematic use—Verbalization	3	7.1%
Self-assessment—Self-knowledge	2	4.8%
Understanding the Origin of the problematic pattern	1	2.4%
Total	42	100.0%

## Data Availability

The data presented in this study are available on request from the corresponding author. The data are not publicly available due to privacy and ethical restrictions.

## Supplementary Materials

The following supporting information can be downloaded at: https://www.mdpi.com/article/10.3390/jcm15124845/s1. Table S1: Individual participant details: prior individual support before joining the twelve-step group; Table S2: COREQ Checklist. Consolidated Criteria for Reporting Qualitative Research.

## Appendix A. Semi-Structured Interview

Thank you for participating in this interview and for having signed the informed consent form. The questions we will discuss aim to understand your personal experiences, perceptions, and thoughts regarding the recovery process. Please, if at any point you feel uncomfortable, do not hesitate to inform me that you prefer not to answer or that you wish to withdraw from the interview.
Section 1: Personal Perception of Recovery
In your own words, what does “recovery” mean to you?
How would you describe your personal experience in the recovery process?
You may mention the most significant aspects or the most important challenges of your journey (What strategies did you use to overcome these challenges?)
Section 2: Accountability and Support
Accountability means building a support relationship through agreed-upon commitments and jointly made decisions. It is being responsible for your actions. What has reporting to other people meant, or what does it mean to you? Do you see it as important within these groups? Has this accountability been important in your recovery?
If so, can you describe that experience and how it impacted your process?
Section 3: Spirituality and Hope
“Spirituality” can be defined as a deep connection with something greater than oneself (God, a higher power…). What place has spirituality held in your recovery process?
Was there any spiritual moment (divine encounter) that particularly stood out and influenced your recovery?
If so, can you describe it and explain its impact?
“Hope”“ is often described as believing in a better future and working to achieve it. How would you describe the role of hope in your recovery?
Is there a specific moment where hope made a significant difference in your process?
If so, what was it and what did you learn from that experience?
Section 4: Participation in Recovery Programs
Some structured programs, such as the 12 steps for sexual addiction, are used in recovery processes. What aspects of that program did you find most useful?
Can you share any prominent experience you had within a structured program that impacted your recovery process?
Section 5: Experiences with Therapies
Have you received individual therapy (psychologist, psychiatrist, therapist…) to try to recover?
If so, for how long?
Have you participated in any other group?
If so, what differences and benefits did you perceive between group therapy and individual therapy?
If you have received both individual and group support, what has each of them provided you?
Can you describe a session—either individual or group—that you found especially helpful?
If a friend were considering therapy for sex or pornography addiction, which would you recommend more: individual or group?
Why do you believe that option would be more suitable?
Conclusion:
Of all the aspects you have mentioned (accountability, spirituality, hope, therapy, structured programs), which do you consider to have been the most important in your recovery process? Why?
Finally, is there anything else you would like to share about your experience that we have not covered in this interview?
Acknowledgement:
Thank you again for sharing your experience. We greatly value your openness and time, and we trust that your perspective will help enrich the understanding of recovery processes.

## References

[1] Cooper A. (1998). Sexuality and the Internet: Surfing into the New Millennium. CyberPsychol. Behav..

[2] Fernández-Conde M.d.M.G., Arquisola C.F., Sánchez L.S., Bravo R.G., Fischer V.J., Alonso M.d.C.F. (2026). El Consumo de Pornografía En Menores y Su Abordaje Desde Atención Primaria de Salud. FMC-Form. Médica Contin. Aten. Primaria.

[3] Weinberg M.S., Williams C.J., Kleiner S., Irizarry Y. (2010). Pornography, Normalization, and Empowerment. Arch. Sex. Behav..

[4] Mehmood Qadri H., Waheed A., Munawar A., Saeed H., Abdullah S., Munawar T., Luqman S., Saffi J., Ahmad A., Babar M.S. (2023). Physiological, Psychosocial and Substance Abuse Effects of Pornography Addiction: A Narrative Review. Cureus.

[5] Bőthe B., Nagy L., Koós M., Demetrovics Z., Potenza M.N., Kraus S.W., International Sex Survey Consortium (2024). Problematic Pornography Use across Countries, Genders, and Sexual Orientations: Insights from the International Sex Survey and Comparison of Different Assessment Tools. Addiction.

[6] (2025). Ministerio de Sanidad Encuesta Sobre Alcohol y Otras Drogas En España (EDADES 2024). Ministerio de Sanidad, Servicios Sociales e Igualdad. https://faecap.es/documentos/tXiIoIz9_XSfQLYQRuD-KmAJl3_18afVmN6AfEQzIGY.pdf.

[7] (2024). Ministerio de Sanidad Encuesta Estatal Sobre Uso de Drogas En Enseñanzas Secundarias (ESTUDES 2023). Ministerio de Sanidad, Servicios Sociales e Igualdad. https://www.sanidad.gob.es.

[8] Voon V., Mole T.B., Banca P., Porter L., Morris L., Mitchell S., Lapa T.R., Karr J., Harrison N.A., Potenza M.N. (2014). Neural Correlates of Sexual Cue Reactivity in Individuals with and without Compulsive Sexual Behaviours. PLoS ONE.

[9] Grubbs J.B., Perry S.L., Wilt J.A., Reid R.C. (2018). Pornography Problems Due to Moral Incongruence: An Integrative Model with a Systematic Review and Meta-Analysis. Arch. Sex. Behav..

[10] Volkow N.D., Koob G.F., McLellan A.T. (2016). Neurobiologic Advances from the Brain Disease Model of Addiction. N. Engl. J. Med..

[11] Antons S., Engel J., Briken P., Krüger T.H.C., Brand M., Stark R. (2022). Treatments and Interventions for Compulsive Sexual Behavior Disorder with a Focus on Problematic Pornography Use: A Preregistered Systematic Review. J. Behav. Addict..

[12] Kelly J.F., Stout R.L., Magill M., Tonigan J.S., Pagano M.E. (2010). Spirituality in Recovery: A Lagged Mediational Analysis of Alcoholics Anonymous’ Principal Theoretical Mechanism of Behavior Change. Alcohol. Clin. Exp. Res..

[13] White W.L. (2007). Addiction Recovery: Its Definition and Conceptual Boundaries. J. Subst. Abus. Treat..

[14] McIntosh J., McKeganey N. (2000). Addicts’ Narratives of Recovery from Drug Use: Constructing a Non-Addict Identity. Soc. Sci. Med..

[15] Cloud W., Granfield R. (2008). Conceptualizing Recovery Capital: Expansion of a Theoretical Construct. Subst. Use Misuse.

[16] White W. (2012). Recovery-Remission from Substance Use Disorders.

[17] Van Manen M. (2003). Researching Lived Experience: Human Science for an Action Sensitive Pedagogy.

[18] Braun V., Clarke V. (2006). Using Thematic Analysis in Psychology. Qual. Res. Psychol..

[19] Marshall C., Rossman G.B. (2015). Designing Qualitative Research.

[20] Krueger R.A., Casey M.A. (2014). Focus Groups: A Practical Guide for Applied Research.

[21] Kvale S., Brinkmann S. (2015). InterViews: Learning the Craft of Qualitative Research Interviewing.

[22] DiCicco-Bloom B., Crabtree B.F. (2006). The Qualitative Research Interview. Med. Educ..

[23] Kalpokaite N., Radivojevic I. (2019). Teaching Qualitative Data Analysis Software Online: A Comparison of Face-to-Face and e-Learning ATLAS. ti courses. Int. J. Res. Method Educ..

[24] Guba E.G., Lincoln Y.S., Denzin N.K., Lincoln Y.S. (1994). Competing Paradigms in Qualitative Research. Handbook of Qualitative Research.

[25] Carrillo Pineda M., Leyva-Moral J.M., Medina Moya J.L. (2011). El análisis de los datos cualitativos: Un proceso complejo. Index Enferm.

[26] Goffman E. (1963). Stigma: Notes on the Management of Spoiled Identity.

[27] Kraus S.W., Voon V., Potenza M.N. (2016). Should Compulsive Sexual Behavior Be Considered an Addiction?. Addiction.

[28] Tangney J.P., Stuewig J., Mashek D.J. (2007). Moral Emotions and Moral Behavior. Annu. Rev. Psychol..

[29] Twohig M.P., Crosby J.M. (2010). Acceptance and Commitment Therapy as a Treatment for Problematic Internet Pornography Viewing. Behav. Ther..

[30] Pagano M.E., Friend K.B., Tonigan J.S., Stout R.L. (2004). Helping Other Alcoholics in Alcoholics Anonymous and Drinking Outcomes: Findings from Project MATCH. J. Stud. Alcohol.

[31] Litt M.D., Kadden R.M., Kabela-Cormier E., Petry N.M. (2009). Changing Network Support for Drinking: Network Support Project 2-Year Follow-Up. J. Consult. Clin. Psychol..

[32] Andersson C., Carlström C., Amroussia N., Lindroth M. (2024). Using Twelve-Step Treatment for Sex Addiction and Compulsive Sexual Behaviour (Disorder): A Systematic Review of the Literature. Sex. Health Compulsivity.

[33] Fernandez D.P., Kuss D.J., Griffiths M.D. (2021). Lived Experiences of Recovery from Compulsive Sexual Behavior among Members of Sex and Love Addicts Anonymous: A Qualitative Thematic Analysis. Sex. Health Compulsivity.

[34] Floyd C.G., Volk F., Flory D., Harden K., Peters C.E., Taylor A. (2022). Sexual Shame as a Unique Distress Outcome of Morally Incongruent Pornography Use: Modifications and Methodological Considerations. Arch. Sex. Behav..

[35] Biernacki P. (1986). Pathways from Heroin Addiction: Recovery Without Treatment.

