# Supplementary material for: Identity Transformation and the Role of Accountability in Recovery from Problematic Pornography Use: A Phenomenological-Hermeneutical Study
Source: J Clin Med. 2026 Jun 22;15(12):4845. doi: 10.3390/jcm15124845 (PMC13302445; doi:10.3390/jcm15124845)
Supplement: Supplementary file 1 [file jcm-15-04845-s001.zip › jcm-4320640-supplementary.pdf]

Table S1. Individual participant details: prior individual support before joining the twelve-step group

P	Type of support	Duration	Perceived efficacy
P1	Spiritual accompaniment + Psychiatric support	4 years	No efficacy: "It didn't work for me at all… I didn't change my habits."
P2	Spiritual accompaniment + Psychological support	~1 year 3 months	No efficacy
P3	Informal (partner) + Psychological support	Not specified	No efficacy: "It was very theoretical and not very practical."
P4	Psychological therapy (unrelated to PPU)	Not specified	Not specified (unrelated to PPU)
P5	Spiritual accompaniment	6 months	Effective: "I started to take more serious steps to put an end to all of this."
P6	No prior individual support	—	—
P7	No prior individual support	—	—
P8	Spiritual accompaniment	10 months	Partial: Helped name the problem but lacked consistent day-to-day feedback
P9	Not specified	Not specified	—
P10	Psychiatric support (multiple professionals)	3–4 years	Not specified
P11	No prior individual support	—	—
P12	Spiritual accompaniment	1 year	Not specified
P13	Psychological support (occasional, post-group)	2–3 sessions	Partial efficacy
P14	Psychological therapy (posterior to process, not PPU-focused)	1 year (ongoing)	Partial / In process
P15	Informal (family) + Spiritual + Psychological + Psychiatric	~1 year 6 months	Partial: "Sometimes I managed to do it for weeks, even months."
P16	Spiritual accompaniment + Psychological support	Not specified	Effective: "It made me realize that I really had a serious problem."
P17	Spiritual accompaniment + Psychological support (trauma-focused)	1 year (psychologist)	Partial: Trauma addressed but PPU not explicitly treated
P18	Psychological + Psychiatric + Therapist support	3–4 years	Partial: "I couldn't have done it only with a psychologist… it really helped me get to know myself better."
P19	Spiritual accompaniment (long-term)	Approx.30 years	Partial: Linked to general personal maturity
P20	No prior individual support	—	—
P21	No prior individual support	—	—
P22	Informal (psychologist friend, telephone) + Self-help	Brief period	No efficacy: "In the end, you get tired of saying it because you fell quite often."
P23	Psychological therapy (unrelated to PPU)	Not specified	Not specified (unrelated to PPU)
P24	No prior individual support	—	—
P25	Psychological + Psychiatric support (multiple professionals)	4–5 years	Partial: Some improvement but PPU persisted
P26	No prior individual support	—	—
P27	No prior individual support	—	—

Note. P = participant code; PPU = problematic pornography use. Support types are self-reported by participants and derived from verbatim interview accounts. Categories are not mutually exclusive. Efficacy ratings reflect participants' own perceptions as expressed during interviews, not standardized clinical measures. Participants P4 and P23 received psychological therapy for issues unrelated to PPU and are therefore not included in the efficacy analysis in the main text.

Table S2. COREQ Checklist. *Consolidated Criteria for Reporting Qualitative Research*

No.	Item	Guide question / description	Reported	Location in manuscript
**Domain 1: Research team and reflexivity**				
1	Interviewer / facilitator	Which author(s) conducted the interview or focus group?	Yes	Author affiliations, p.1
2	Credentials	What were the researcher's credentials, e.g. PhD, MD?	Yes	Author affiliations, p. 1
3	Occupation	What was their occupation at the time of the study?	Yes	Author affiliations, p. 1
4	Gender	Was the researcher's gender specified?	Yes	Section 2.2 — male interviewer
5	Experience and training	What experience or training did the researcher have?	Yes	Section 2.1 — phenomenological-hermeneutical design (Van Manen)
6	Relationship with participants	Was a relationship established prior to study commencement?	Yes	Section 2.2 — no prior friendship; participants were aware of the researcher's presence at some meetings before being interviewed
7	Participant knowledge of the interviewer	What did participants know about the researcher, e.g. personal goals, reasons for doing the research?	Yes	Introduction — research gap in subjective recovery experience
8	Interviewer characteristics	What characteristics were reported about the interviewer / facilitator, e.g. bias, assumptions, reasons and interests in the research topic?	Yes	Section 2.1 — phenomenological bracketing and hermeneutical framework
**Domain 2: Study design**				
9	Methodological orientation and theory	What methodological orientation was stated to underpin the study, e.g. grounded theory, discourse analysis, ethnography, phenomenology, content analysis?	Yes	Section 2.1 — van Manen hermeneutical phenomenology
10	Sampling	How were participants selected, e.g. purposive, convenience, consecutive, snowball?	Yes	Section 2.2 — purposive sampling (defined inline)
11	Method of approach	How were participants approached, e.g. face-to-face, telephone, mail, email?	Yes	Section 2.2 — contact made through group coordinators and meetings
12	Sample size	How many participants were in the study?	Yes	Section 2.2 — N=27
13	Non-participation	How many people refused to participate or dropped out? Reasons?	Yes	Section 2.2 — 34 contacted, 27 agreed to participate; 7 declined (acceptance rate 79.4%)
14	Setting	Where was the data collected, e.g. home, clinic, workplace?	Yes	Section 2.2 — face-to-face interviews at meeting venues or locations agreed with participants

No.	Item	Guide question / description	Reported	Location in manuscript
15	Presence of non-participants	Was anyone else present besides the participants and researchers?	Yes	Section 2.2 — individual interviews, no third parties present
16	Description of sample	What are the important characteristics of the sample, e.g. demographic data, date?	Yes	Section 2.2 and Table 2
17	Interview guide	Were questions, prompts, guides provided by the authors? Was it pilot tested?	Yes	Appendix A — interview guide; Section 2.2 — pilot interview conducted with group coordinator (not included in analysis)
18	Repeat interviews	Were repeat interviews carried out? If yes, how many?	N/A	Single interview per participant
19	Audio / visual recording	Did the research use audio or visual recording to collect the data?	Yes	Section 2.2 — audio-recorded and transcribed verbatim
20	Field notes	Were field notes made during and / or after the interview or focus group?	No	Not collected; audio recording and verbatim transcription were used as primary documentation
21	Duration	What was the duration of the interviews or focus group?	Yes	Section 2.2 — mean duration approximately 25 minutes
22	Data saturation	Was data saturation discussed?	Yes	Section 2.2 — thematic sufficiency (information power) reached between P24 and P27
23	Transcripts returned	Were transcripts returned to participants for comment and / or correction?	Yes	Section 2.3 — results were shared with several participants who asked about the study; no corrections were reported
**Domain 3: Analysis and findings**				
24	Number of data coders	How many data coders coded the data?	Yes	Section 2.3 — two coders (co-authors)
25	Description of the coding tree	Did authors provide a description of the coding tree?	Yes	Section 2.3 and Table 3 — 151 codes, 9 categories, 4 metacategories
26	Derivation of themes	Were themes identified in advance or derived from the data?	Yes	Section 2.3 — inductive coding; themes derived from the data
27	Software	What software, if applicable, was used to manage the data?	Yes	Section 2.3 — ATLAS.ti
28	Participant checking	Did participants provide feedback on the findings?	Yes	Section 2.3 — a posteriori, several participants asked about results; partial findings were shared informally
29	Quotations presented	Were participant quotations presented to illustrate the themes / findings? Was each quotation identified, e.g. participant number?	Yes	Section 3 — verbatim quotations throughout, identified by P1–P27 codes
30	Data and findings consistent	Was there consistency between the data presented and the findings?	Yes	Section 2.3 — triangulation via three-level cyclical analysis; credibility criteria of Guba and Lincoln

No.	Item	Guide question / description	Reported	Location in manuscript
31	Clarity of major themes	Were major themes clearly presented in the findings?	**Yes**	Section 3 and Figure 1 — interpretive model with three phases and sub-themes
32	Clarity of minor themes	Is there a description of diverse cases or discussion of minor themes?	**Yes**	Section 4.8 — contrast with existing literature; divergent cases noted (2 participants declined after initial contact)
